# Professional Changes

**Published:** 1856-07

**Authors:** 


*Professional Changes.*

Thomas D. Mutter, M.D., for many years the able Professor of Surgery in Jefferson Medical College, Philadelphia, has resigned on account of failing health; and Prof. S. D. Gross, of Louisville, Ky., has been appointed to fill the vacancy.

Prof. A. Flint has resigned the Chair of Practice in the University of Louisville, and accepted the Chair of Clinical Medicine and Pathology in connection with the Medical Department of the University of Buffalo, where he has long resided.

Prof. H. A. Ackley, who has occupied the Chair of Surgery in the Cleveland Medical College, since the organization of the School, has resigned.

